# Misuse of emergent healthcare in contemporary Japan

**DOI:** 10.1186/s12873-017-0135-4

**Published:** 2017-07-14

**Authors:** Yasuhiro Kadooka, Atsushi Asai, Aya Enzo, Taketoshi Okita

**Affiliations:** 1 Department of Bioethics, Kumamoto University Faculty of Life Sciences, 1-1-1 Chuo-ku Honjo, Kumamoto City, 8608556 Japan; 2 Department of Medical Ethics, Tohoku University Graduate School of Medicine, 2-1 Aoba-Ku Seiryomachi, Sendai, 9808575 Japan

**Keywords:** Medical misuse, Emergency care, Medical resources, patient’s duty

## Abstract

**Background:**

Medical care is obviously an important public service to ensure the health of a nation; however, medical resources are not always used appropriately. ‘Convenience-store consultations’ and inappropriate ambulance transportation represent instances of such improper use by contemporary Japanese citizens in recent years. This article illustrates two examples of misuse and discusses potential countermeasures by considering factors contributing to these behaviours.

**Main body:**

From both public and medical perspectives, these patient behaviours are problematic, causing potential harm to others, negative consequences to such patients themselves, exhaustion of healthcare staff, and breakdown of emergency medical services. Although citizens need to recognize the public nature and scarcity of medical care, the more immediate need may be to identify and to remove personal and social causes inducing such misuse. In addition, healthcare professionals should become more trustworthy. To combat these issues, one-sided penalties such as accusations or sanctions for patients who misuse the system cannot be justified in principle. If measures taken to prevent misuse are ineffective, imposing surcharges or restricting consultations may be considered official policy, but these are not acceptable for several reasons.

**Conclusion:**

For now, we conclude that we must rely on the spontaneous motivation of patients who engage in ‘convenience-store consultations’ and ambulance transportation instead of taking a taxi.

## Background

Healthcare is interpreted as a part of social security based on Article 25 of Japan’s Constitution [1]. The fundamental ideal is fairness; Japan adopted a universal healthcare system in 1961 that guarantees patients free access to medical care. Generally, citizens who have a health insurance policy can consult the facility and doctor of their choice at any time and as many times as they want. However, free access might have led to some negative effects. Mildly-ill patients skip consulting primary care physicians and directly access emergency rooms of higher local base hospitals which assume a role to provide secondary or tertiary medical care, increasing the burden on ER medical staff [2]. In addition, maldistribution of physicians has been a serious issue in Japan, with many regions facing a doctor shortage [3]. Therefore, a healthcare crisis exists especially in physician-shortage regions, and this includes exhaustion, burnout, and increasing turnover among healthcare professionals, as well as recent hospital and ward closures [4]. A more appropriate distribution of physicians, however, is not the only solution to these problems. One writer has argued that the current universal healthcare system in Japan, which guarantees free access to medical facilities, will become insolvent in the future [5].

In addition to protecting and securing human resources in medical facilities and public administrations, attention should be directed to patient attitudes; however, this issue has not provoked ethical debate. We consider the patients’ ‘convenience-store consultation’ and transportation by ambulance instead of a taxi as recently identified factors contributing to the healthcare crisis. These two patient behaviours are clearly problematic from both public and medical healthcare perspectives. In addition to an increasing frequency of ambulance mobilization annually in Japan [6], healthcare professionals including ambulance crews are imposed an obligation to provide fundamental services by relevant laws and administrative guidelines, and their refusal to patients’ request is accepted under very limited circumstances, even with justifiable reasons. Therefore, the two problematic patient behaviours potentially exacerbate physical and emotional exhaustion of healthcare professionals in emergency medicine, potentially leading to declines in patient care. Another serious result anticipated is the breakdown of the healthcare system. In fact, healthcare facilities in several regions of Japan have been in danger of closing several treatment divisions because high ‘convenience-store consultation’ caseload have exhausted healthcare professionals [7]. With medical cost inflation in Japan, a major newspaper advocated for changing civic awareness based on reported cases of such healthcare misuse [5]. This article will highlight these newly emerged Japanese healthcare issues that have not been discussed in detail, probe relevant causes, and consider measures to be taken. In addition, it will discuss the pros and cons of restricting patient access to healthcare or charging additional consultation fees. Finally, it will show that there are no effective measures to be taken at this stage for the misuse of healthcare, other than relying on patients’ spontaneous motivation.

### ‘Convenience-store consultations’

One recent healthcare misuse in Japan is the so-called ‘convenience-store consultation,’ which is defined by a patient’s casual use of secondary or tertiary emergency outpatient services even when their symptoms and conditions are mild [8]. These patients with common colds and scrapes may notice symptoms during weekday daytime hours, but choose to visit the hospital after office hours, reasoning that, “There is less of a wait at night,” “I had business appointments during the day,” and so on. This affects after-hours emergency medical services at the hospital, because emergency medical staff cannot concentrate on caring for critically ill patients [9, 10]. A few studies have examined this situation. In one survey, 97 of 190 patients who visited a secondary emergency room were judged as ‘convenience-store consultation’ cases [11]. In another survey, an average of 90 patients per day visited an emergency room at a tertiary care centre between January and October 2014, and nearly 70 per day were considered mild cases [10]. Another article reported that 14.2% of after-hours patients in May 2009 had noticed symptoms during the day [12]. The secondary care hospital engaged in public awareness activities to reduce such consultations, but the rate was still 11.9% in May 2010. Responding to seemingly ineffectiveness of the activity, authors of the article commented that wide-ranging efforts with the assistance of local administration, medical associations and mass media would be necessary [12].

Essentially, emergency medical services target a limited number of critically ill patients who need urgent treatments. Some patients who engage in ‘convenience-store consultation’ request specialists and detailed examinations in addition to reasonable primary care. Healthcare staff facing such patients may struggle to dissuade them, and increases in such cases affect care for severely ill and hospitalized patients. Doctors are exhausted and daytime medical practice in the hospital can be compromised [9]. A physician-writer bemoaned the situation, saying “All patients believe that they undergo cutting-edge medical care and get cured immediately by visiting the emergency room [13].” In one region, there were public campaigns calling to withdraw ‘convenience-store consultation’, to visit doctors within regular business hours, and to give priority to seriously ill patients [9]. In addition, many hospitals began to collect surcharges equally from all after-hours visiting patients [14]. For example, a newspaper reported that a hospital charged 5400 JPY over the net medical expenses to patients who requested medical care even if they did not need urgent care; this caused a gradual decrease in casual hospital visits [2].

### Using an ambulance like a taxi

The other problematic behaviour in contemporary Japanese healthcare is the use of an ambulance instead of a taxi to visit emergency rooms. The Japanese Ministry of Internal Affairs and Communication reported that there were almost 6 million cases of ambulance mobilization in 2014 and that 49.4% of them were judged to be for minor conditions. The number of ambulance mobilizations in Japan increased by 31% in those ten years [15]. The Ministry warned of the possible delay in urgent treatment for patients who truly need it, as well as a potential decrease in the rate of patient survival [16]. Similar to those who engage in convenience-store consultations, patients who casually call for an ambulance had mild conditions, which even included mosquito bites, sunburn irritation, jammed fingers, incision wounds and so on. [16] An emergency service office reported patient reasons for inappropriate ambulance usage, including intolerance for waiting times during the day, not owning a private motor vehicle, and the cost of a taxi ride. There was even a case in which a patient asked for a ride home [17]. Some patients who should have visited their primary care physicians or ask for home-visiting nurses have instead called for an ambulance [4].

The National Centre for Child Health and Development reported rates of ambulance-transported paediatric patients between 2011 and 2012 who were triaged as non-urgent and returned home without any examination or treatment [18]. The authors of this report found that the number of non-critical paediatric patients increased between 5 pm and midnight compared to other time periods, and that patients who could be cared for at non-emergency facilities used an ambulance for transportation [18]. Another hospital reported that 128 of 331 cases (42.8%) of ambulance-transported patients were not hospitalized. Some medical professionals who engaged in the urgent activities responded that patients were in little need of ambulance transport [19]. Reasons for calling an ambulance included wishes of patients or their families to be transported by ambulance assuming that their conditions were serious; lack of transportation means; hope for receiving prompt medical care; and instructions from primary care physicians [19]. The authors of the article highlighted the necessity for triage before mobilization, including telephone consultation and education not only to citizens, but also to office-based physicians who casually recommend ambulance transportation [19]. They also insisted that the Japanese free ambulance transportation service was a factor of such thoughtless usage [19].

### Problems with convenience-store consultations and using ambulance transportation instead of taxis

These behaviours that misuse emergency medical services share several problems in common. The first is the potential harm to other patients, citizens and the regional medical care system. These inappropriate behaviours may prevent patients who truly need immediate and critical care from being treated suitably. As the number of mobilizations increases, more time is needed for the ambulance to reach the patient [20]. Additionally, care for ‘convenience-store consultation’ patients interrupts emergency room medical staff from initiating treatment for patients who require it immediately. Mildly ill patients who occupy ambulance services, beds and physicians in the emergency room can deprive other patients from potentially needed urgent and life-saving therapies.

Second, patients who engage in convenience-store consultations or who use an ambulance like a taxi may also harm themselves. Physicians who treat patients at emergency rooms are not the primary care doctors of these patients; in fact, they are unlikely to have a long, stable relationship with the patient or any prior knowledge of their case. The primary role of the emergency division after hours is not to perform specialized medical tests and treatments, but triage and life-saving treatments. Thus, the treatment goal may not be effectively fulfilled because time-limited physicians cannot identify definite and sufficient examinations and treatments, the hospital cannot provide them at the time, and the patient does not receive them in a timely manner. In addition, patient payments may increase. Inappropriate emergency room visits are simply not the best medical behaviour for such patients.

Third, if we presume that patients should take responsibility for healthcare access, including emergency medical services, then ‘convenience-store’ consultations and inappropriate ambulance transportation clearly represent an unfair use of the public funding. The contemporary Japanese universal healthcare insurance coverage system, which allows citizens to receive medical services of a certain standard at every medical facility by only offering their healthcare insurance card, has earned admiration. However, the healthcare system has limited finances and manpower, and these resources are not secure enough to tolerate the inappropriate use of the system. It is possible to argue that patients must respect the public funding to ensure that other patients and even they themselves are not prevented from receiving medical treatment when it becomes necessary. The question has recently emerged as to how many in the Japanese public recognize the preciousness of the system [1]. Patients misusing the medical system are essentially neglecting their duties. Usage of emergency medical services based only on one’s own convenience is an abuse of a limited societal resource. If a patient recognizes that healthcare is a finite and important social resource, and that healthcare professionals comprise elements of this resource, he/she would not make casual visits to an emergency room on the way home from work or use an ambulance as a mere means of transportation [21].

### Investigating the causes of emergency medical care misuse as a prioritized measure

Certainly, the first priority to control ‘convenience-store’ consultation and inappropriate ambulance transportation is identifying the causes of such healthcare misuse. It is very important to clarify whether they are issues of individual morality or not. This may necessitate an investigation of the socioeconomic status of patients who are prone to misusing the healthcare system, frequented areas, and attitudes of other citizens and healthcare professionals toward the use of emergency medical care. We have collected as many relevant writings as possible, but the causes remain unclear. One newspaper surveyed patients who visited a university hospital emergency room at night [22]. Nearly half of patients who required medical care had symptom onset during the daytime when usual care is provided. Work pressures including job duties, reluctance to ask permission from superiors, and having no paid holidays were among reasons for their night visit to the ER [22]. The newspaper article suggested that a patient’s work environment influences their behaviour, including hospital consultation, and that the Japanese culture of overworking led to inappropriate use of emergency care [22]. Patients working busy schedules under strong pressure from superiors or those with a strong sense of responsibility might be sensitive to their professional demands, feeling that they cannot consult doctors during regular office hours. Such patients are likely to engage in ‘convenience store consultations.’

This does not seem to be the only cause. Many citizens do not possess sufficient knowledge of the system involving medical care and its various services. They may not know that emergency medical services are functionally and systematically different from ordinary medical care. Because of this lack of knowledge, patients cannot calmly discern whether they are in a mild or severe condition. Even if the condition is not severe, they may subjectively experience intense fear or anxiety so that they ask for emergency care or even call for an ambulance. If a frail patient’s condition changes a little, the family may want to ask for a medical evaluation and treatment. It may be too demanding for childrearing parents to make level-headed judgments about the health of their children, and financially challenged patients who are orthopedically impaired or live a great distance from hospitals cannot easily take a taxi to consult with a physician. Patients who lack sufficient knowledge about healthcare may head to ER or call ambulance service casually.

Currently, definitive causes for the misuse of emergency medical services remain unidentified. Further close investigation of both individual and social factors is needed in order to control the two problematic behaviours. At this time, it is unsuitable to blame patients who commit such healthcare misuse as having individual moral deficits. Direct interventions to such patients are not the only requirement. Many factors should be considered for each patient, including working environment, economic status, family support, and the medical system in their area. It is too early to question and reprimand with indignation the patient who visits an emergency room for a mild condition. We should examine where individual responsibility ends and social problems begin.

### Possible countermeasures

There seem to be several ways to prevent the collapse of emergency medical services in Japan in addition to investigating the causes of the misusing behaviours. First, citizens should choose their own primary care doctor. One poll reported that patient respondents who consulted regularly with a particular physician were more readily able to regulate their daily lives and attend check-ups [23]. If patients increase their interest and knowledge in health and medical care, they become better able to consult doctors appropriately. Introduction of primary care doctor system by public administration may be an effective measure to control ‘convenience-store consultation’ and unnecessary ambulance transportation. Also, healthcare professionals should make an effort to be trusted by citizens so that patients will wish to visit their regular doctors to receive medical care. Few patients consult untrustworthy medical facilities. Healthcare professionals should fulfil their duties of patient education and self-improvement as well, which results in lower incidences of inappropriate behaviour among patients, including ‘convenience-store consultation’ and unnecessary ambulance transportation.

Second, citizens should be empowered by public administration to make appropriate medical judgments regarding consultation. For example, in the UK, the NHS Primary Care Trust conducted a behaviour analysis of patients in an area with a high number of ‘convenience-store consultations’ and reported that more than half of them had insufficient knowledge of disease and medical care. Subsequently, the public service developed an education program and has provided a telephone consultation service to avoid urgent consultation by patients who are not severely ill [24]. Patients should be provided with the proper quantity and quality of medical knowledge repeatedly, continuously, and in a timely manner. With increased proper relevant advice and instruction, they can become competent enough to make appropriate judgments on the use of emergency medical care.

Third, a public enlightenment program to increase citizen awareness regarding the public nature and scarcity of healthcare may be effective. In Japan, because the population is aging and there is a decrease in the number of those of reproductive age, it has become difficult to secure financial resources for healthcare. Public campaigns of enlightenment have already been launched and should be run tirelessly until citizens realize the value of the current universal healthcare system, which provides high access to and basic freedom of medical care. This campaign should also raise awareness that healthcare misuse leaves them worse off.

### Surcharges and restriction of medical care

We must avoid situations that prevent the maintenance of the current medical system that allows free access to medical care. Simultaneously, fellow citizens and patients must not be regarded as perpetrators who rob others of health, including the timely and adequate access to medical care. Healthcare misuse should be minimized. Notably, any intervention proposed above may not work as effectively as expected. Possible measures that can be quickly implemented by medical facilities or public administrations may include imposing a surcharge or restricting public funds to patients who are considered to misuse the system. For example, a system in which patients with mild conditions must pay a surcharge for each inappropriate ambulance transportation and ‘convenience-store consultation,’ or a flat-fee system where only patients with severe conditions are refunded later can be imagined. There already have been several facilities that have deployed the former measure [2].

That said, such measures may also need to be avoided due to the lack of fair standards for medical judgment and the likelihood that they would provoke certain side effects. First, we do not have universally accepted medical standards for disease severity. Diseases and patient conditions vary widely in actual clinical settings. They must make judgments based on many variables, which sometimes include psychological and social aspects. They may be biased against alcoholic or homeless patients, or welfare recipients according to individual conscience and values. There is a quite wide variety of actual situations, which makes it difficult to make a prompt decision about the suitability of a patient’s ambulance call and emergency room consultation. Therefore, uniform standards seem unfair and unacceptable. Even if any uniform standard is established, some patients who are excluded from it may face a life-threatening crisis. This is probably an infringement on one’s right to life.

Second, such a decision concerning the suitability of a patient’s consultation may be burdensome for healthcare professionals. Additional office hours are required and compassionate healthcare staff may bear a psychological burden. There is also the possibility of judgment errors. In such cases, healthcare staff members need additional time to respond to adverse claims from patients and to reimburse them. Emergency rooms, which are usually busy, probably face even greater chaos.

Third, if the surcharge amount is set at a high level, patients with financial difficulties may avoid using the ambulance and emergency room services, even if they are in a critically severe condition, and thus would be more likely to be deny themselves appropriate and necessary treatments. This situation can lead to health disparities, which contradicts the fundamental ideal underlying the contemporary Japanese universal healthcare system. Conversely, if the amount is set at a low level, the restraining effect would be limited. Many patients with adequate finances would repeat abusive healthcare behaviours, which may prevent treatment for critical patients and exhaust healthcare professionals in the emergency room. Additionally, as regional income levels differ, nationally uniform standards would be unfair. Surcharges and a flat-fee system are not feasible.

Fourth, restriction of emergency medical treatment by stigmatization or putting such patients on a misuser blacklist is possible options. A physician has surmised that healthcare misuse has increased as a result of a growing sense of the civic rights for self-determination as well as access to administrative services [25]. This view may support patients consuming as they wish, as long as they pay. Based on these rights, some people may never change their ways even if requested to do so by medical or administrative services. However, all patients should recognize that the healthcare system relies upon solidarity, and that a citizen’s duty is to maintain it; unfortunately, some are habitual healthcare abusers, violating civic virtue. It may be effective to restrict their ambulance use and emergency room visits, but there may be negative consequences, like the Boy Who Cried ‘Wolf’, when they are truly experiencing severe symptoms. Alternatively, they may seek other medical facilities for treatment. Therefore, stigmatization and blacklists can infringe upon one’s right to life and may involve arbitrary or discriminatory judgments by healthcare professionals. When a patient who is misusing the system is severely injured because of treatment or consultation restriction, the positive effects of these measures do not seem to balance out the negative.

## Conclusion

A universal healthcare system that allows citizens free access to any treatment and ambulance services has provided Japanese citizens with peace of mind and life-saving care [26]. Possible effective measures to control patients’ misuse of emergent healthcare include having primary care physicians, physicians’ effort to be trusted by their patients, public administration services to educate citizens, telephone services to provide appropriate medical information, and public enlightenment campaigns regarding the limitation and sustainability of universal healthcare system. In addition, surcharges and restricted access for emergency medical services could be considered but are unacceptable and not ideal due to several practical issues, given the wide variety of patient conditions in actual settings and potential of rights to fair access to healthcare and life. In order to prevent emergent healthcare from collapsing, we must seek more effective measures, which remain undiscovered. Thus, it seems there is no other choice but to rely upon spontaneous ethical motivation. At the same time, compelling questions remain unresolved, and include the degree of citizen responsibility for maintaining the current healthcare system and the extent of seeking self-responsibility for individual acts. The prioritized measure is to foster citizen awareness of these problems. For this purpose, we should probe the current causes of healthcare misuse and establish trustworthy healthcare services. Subsequently, we should discuss and build a consensus concerning the patient’s duty as a citizen and then must help to increase citizen awareness surrounding the public nature and scarcity of healthcare resources. Healthcare professionals are not justified in expressing righteous indignation to patients misusing the medical system for now.
